# The value of chest computed tomography in evaluating lung cancer in a lobe affected by stable pulmonary tuberculosis in middle-aged and elderly patients: A preliminary study

**DOI:** 10.3389/fonc.2022.868107

**Published:** 2022-10-06

**Authors:** Kui Long, Hui Zhou, Yajuan Li, Liang Liu, Jiahui Cai

**Affiliations:** ^1^ Department of Radiology, Xiangya Hospital, Central South University, Changsha, China; ^2^ Department of Radiology, Xiangya Changde Hospital, Changde, China; ^3^ National Clinical Research Center for Geriatric Disorders, Xiangya Hospital Central South University, Changsha, China; ^4^ Department of Radiology, Qingyuan people’s Hospital, Qingyuan, China

**Keywords:** lung cancer, pulmonary tuberculosis, computed tomography, follow-up, misdiagnosis

## Abstract

**Introduction:**

Lung cancer can be masked by coexisting stable tuberculosis lesions, which may result in delayed lung cancer diagnosis and treatment. Information about pulmonary tuberculosis patients who are at high-risk of developing lung cancer is scarce. We aimed to examine the value of chest computed tomography (CT) in evaluating lung cancer in a lobe affected by stable pulmonary tuberculosis in middle-aged and elderly patients.

**Methods:**

In this single-centered, retrospective, observational study, we enrolled 41 middle-aged and elderly patients with pulmonary tuberculosis who developed lung cancer in the same lobe from January 30, 2011 to December 30, 2020. Comparisons of the clinical and chest CT data were made with age-matched and sex-matched control groups of patients with stable pulmonary tuberculosis but no lung cancer diagnosis (n = 38).

**Results:**

Seventeen patients in the lung cancer group (41%) were initially misdiagnosed. Compared to lesions in the control group, lesions in the lung cancer group were significantly more likely to demonstrate the following CT features: large size, vessel convergence, lobulation, spiculation, spinous protuberance, bronchial obstruction or stenosis, vacuolation, ground-glass opacification, heterogeneous or homogeneous enhancement, and gradual increase in size. Nodular enlargement showed the best diagnostic performance in the diagnosis of lung cancer in a lobe affected by tuberculosis (area under the receiver operating characteristic curve = 0.974; P <0.001; accuracy = 98.2%; sensitivity =94.7%; specificity = 100%).

**Conclusion:**

Chest CT might play an important role in early diagnosis of lung cancer in a lobe affected by tuberculosis. Regular CT re-examination is necessary in continuous controls monitoring of patients with stable pulmonary tuberculosis. The study indicates necessity of prospective study in this field.

## Introduction

Although the current coronavirus disease 2019 (COVID-19) pandemic has caused considerable illness and death around the world since early 2020, the impact of tuberculosis has been even greater. Tuberculosis remains a widespread disease in many developing countries, including China, and more than 1.4 million people worldwide die of tuberculosis every year (1). Among all cancers in China, lung cancer has the highest prevalence and causes the greatest number of deaths (2). In addition, studies have shown that lung cancer is more likely to develop in pulmonary tuberculosis patients than the general population (3, 4). Lung cancer can be masked by coexisting stable tuberculosis lesions in the same lobe, which may result in delayed lung cancer diagnosis and treatment as well as worse patient outcomes. In this retrospective case-cohort study, we analyzed and compared clinical and computed tomography (CT) data of all consecutive patients with pulmonary tuberculosis who developed lung cancer with age- and sex-matched controls (pulmonary tuberculosis patients without lung cancer) to investigate CT predictors of lung cancer. Our aim was to examine the value of CT screening in pulmonary tuberculosis patients who are at high-risk of developing lung cancer.

## Materials and methods

### Study design and patients

**Figure 1:**
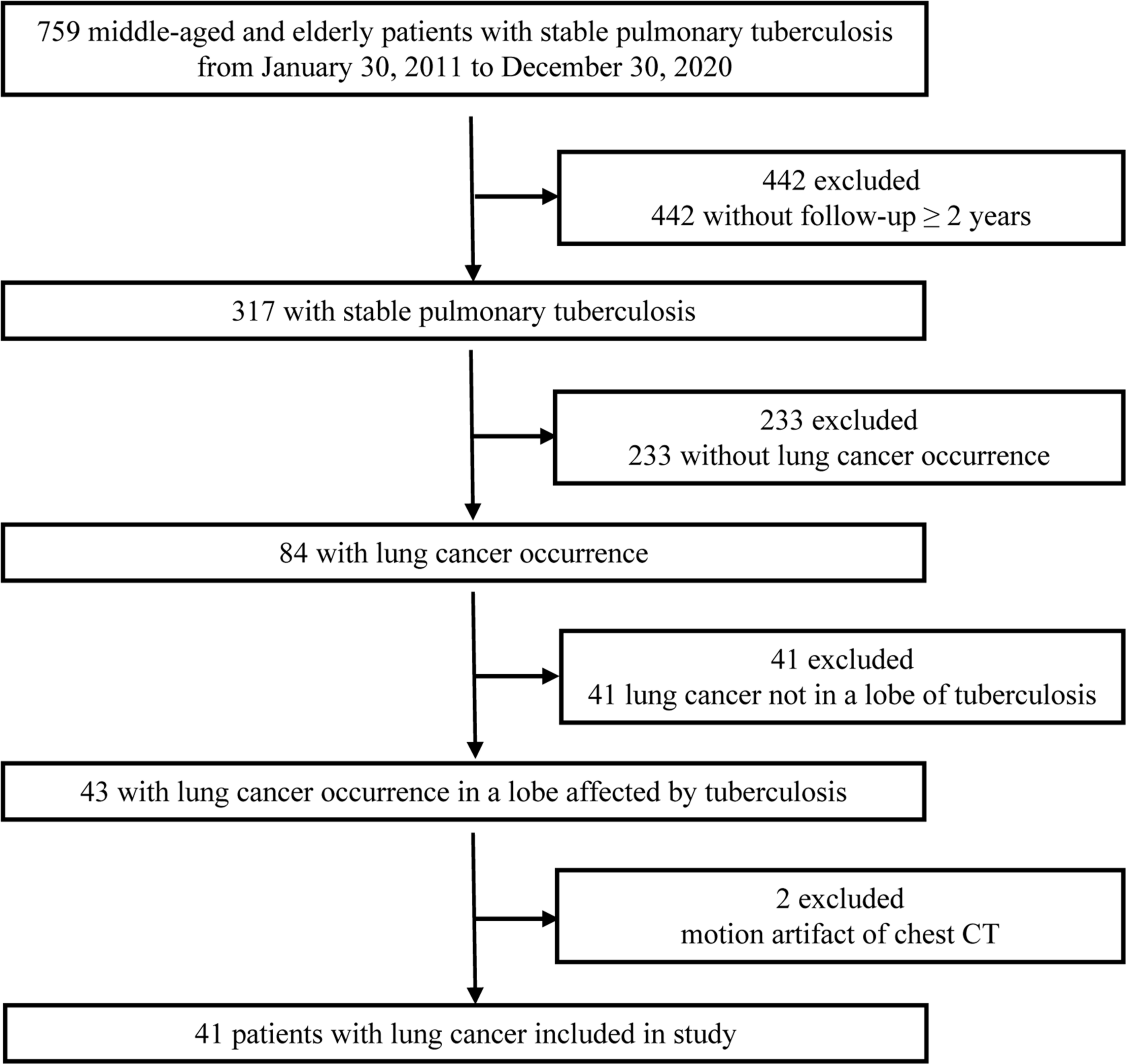
Study flow diagram CT, computed tomography.

The clinical and chest CT data of 759 consecutive middle-aged and elderly patients with stable pulmonary tuberculosis diagnosed in our hospital from January 30, 2011 to December 30, 2020 were analyzed retrospectively. The study was approved by our Medical Ethics Committee (No. 202012230). Stable tuberculosis was identified by comprehensive analysis of clinical manifestations, treatment history and imaging (5). The presence and localization of pulmonary tuberculosis was determined on chest CT by two radiologists (with 5 and 19 years of chest CT interpretation experience, respectively) who reached a consensus regarding tuberculous lesion location and activity. All the included patients were followed up for more than 2 years and diagnosed with lung cancer in the same lobe of tuberculosis. 718 patients (95%) were considered ineligible, including two patients who had motion artifact of chest CT. 41 patients with lung cancer (5%) occurrence in a lobe affected by tuberculosis were included in this study (**Figure 1**). Histopathological confirmation of lung cancer (including secondary scar carcinoma and primary cancer) was obtained by percutaneous biopsy, bronchoscopic biopsy, or surgical resection. In the lung cancer group, the pathological types were as follows: adenocarcinoma, 26 cases; squamous cell carcinoma, 8 cases; unclassified non-small cell lung cancer, 4 cases; small cell lung cancer, 2 cases; and sarcomatoid carcinoma, 1 case. Comparisons were made with age-matched and sex-matched control group of patients with stable pulmonary tuberculosis but no lung cancer diagnosis (n = 38). The detailed information of control group was listed in **Table 1**. 22% of patients of lung cancer group (9/41) underwent PET/CT after finding the suspected malignant lesions by CT before surgery, while none of the control group underwent PET/CT.

**Table 1:** Clinical data of patients with lung cancer occurrence in a lobe affected by tuberculosis and control group.

Project	Normal range	Lung cancer group (n = 41)	Control group (n = 38)	*t/χ²* value	*P* value
Age (years)		66.2 ± 8.4	65.8 ± 12.0	0.161	0.872
SexMaleFemale		34 (83%)7(17%)	27 (71%)11(29%)	1.561	0.212
Years of tuberculosis		16.9 ± 15.3	11.9 ± 11.9	1.115	0.272
Smoking		26 (63%)	19 (50%)	1.429	0.232
Diabetes		3 (7%)	5 (13%)	0.730	0.393
CEA, elevated	< 5.0 ng/mL	13 (13/34, 38%)	0 (0/9, 0%)	4.325	0.038
NSE, elevated	< 13.0 ng/mL	1 (1/33, 3%)	1 (1/22, 5%)	0.085	0.771
CA-125, elevated	< 35.0 KU/L	4 (4/23, 17%)	0 (0/14, 0%)	2.539	0.111
SCC, elevated	< 1.2 ng/mL	1 (1/19, 5%)	0 (0/9, 0%)	0.474	0.491
NSCLC-21-1, elevated	< 3.0 ng/mL	3 (3/18, 17%)	0 (0/9, 0%)	1.625	0.238
CK19, elevated	< 4.0 mU/ml	4 (4/33, 12%)	1 (1/15, 6%)	0.322	0.570
Tuberculosis antibody, positive		4 (4/27, 14%)	3 (3/15, 18%)	0.229	0.633
Tuberculin skin test, positive		18 (18/33, 55%)	13 (13/22, 59%)	0.109	0.741
TSPOT, positive		20 (20/28, 71%)	10 (10/12, 83%)	0.619	0.431

### CT and imaging interpretation

CT was performed using a 256-row (Revolution CT; General Electric, Boston, MA, USA) or 64-layer dual source (SOMATOM Definition; Siemens, Munich, Germany) scanner. Patients were scanned in the supine position from the top of the lung to the diaphragm during breath holding. The SOMATOM Definition main technical parameters were: 100kV, 150 mA, pitch value 0.6–0.9, and scanning layer thickness 1 mm. The Revolution CT main technical parameters were: 120 kV, 80–350 mA, pitch value 0.992, scanning layer thickness 0.625 mm, reconstruction thickness 1 mm, reconstruction interval 1 mm, reconstruction matrix 512 × 512. Some patients underwent a contrast-enhanced examination with a delay of 60 seconds. Non-ionic contrast medium (Ultravist; Bayer, Leverkusen, Germany) with iodine concentration of 300 mg/mL was injected into an antecubital vein at a rate of 2.0 mL/s for a patient dose of 1.5 mg/kg.

All the first chest CT scans after the diagnosis of stable pulmonary tuberculosis were subject to blind review by two radiologists mentioned above in consensus. If the results were inconsistent, a consensus was reached after negotiation. All images were viewed on both the lung (width, 1200 HU; level, − 600 HU) and mediastinal (width, 350 HU; level, 40 HU) settings on the picture archiving and communication system. The following tuberculosis lesion characteristics (6, 7) were recorded: A) morphological characteristics (length and diameter of nodules, vessel convergence, lobulations, spiculations, spiculation protuberance, bronchial stenosis or occlusion, obstructive pneumonia, obstructive atelectasis, necrosis, cavitation, vacuolation, calcification, presence of ground glass nodules and/or satellite foci); B) non-enhanced CT value and enhancement characteristics of tuberculosis nodules, including minimum CT value, average CT value, degree of nodular enhancement, and enhancement pattern; C) characteristics of enlarged mediastinal lymph nodes; D) pleural thickening; E) lesion size on follow-up examination. To obtain the proper CT value (8), the region of interest (ROI) of target lesion was cautiously drawn freehand around the peripheral boundary of the lesion to exclude surrounding air, blood vessels, and atelectatic lung. The ROI was drawn as large as possible to minimize influences of noise and the partial volume effect, and calcified areas were avoided as far as possible. The selected sections of measurement remained the same before and after enhancement. If there was substantial artifact from respiration or cardiac motion, the image was eliminated from data analysis. The definition of nodular enlargement is an increase in size of > 1.5 mm (> 2 mm^3^) measured at baseline and during follow-up based on Lung‐RADS Version 1.1 (9).

### Statistical analysis

Statistical analyses were performed using SPSS software version 23.0 (IBM Corp., Armonk, NY, USA). Categorical variables are presented as numbers with frequency and were compared using the *χ²* test or Fisher’s exact test. Continuous variables are presented as means with standard deviation and were compared using one-way analysis of variance or the independent-samples t-test as appropriate. Receiver operating characteristic (ROC) curves were created using MedCalc software version 18.2.1 (MedCalc Software, Ltd., Ostend, Belgium) and the area under the curve (AUC) was calculated. The cut-off value was obtained using the Youden index method. Sensitivity, specificity, and accuracy were calculated. *P <*0.05 was considered significant.

## Results

### Clinical characteristics and laboratory findings

Patient clinical characteristics and laboratory findings are shown in **Table 1**. Serum CEA concentration was significantly higher in the lung cancer group than the control group (*P* = 0.038). Patient age; sex; smoking; diabetes; years of tuberculosis; concentrations of NSE, NSCLC-21-1, CA 125, SCC antigen, and CK-19; and tuberculosis antibody and T-SPOT testing did not significantly differ between the two groups.

### CT characteristics

**Figure 2:**
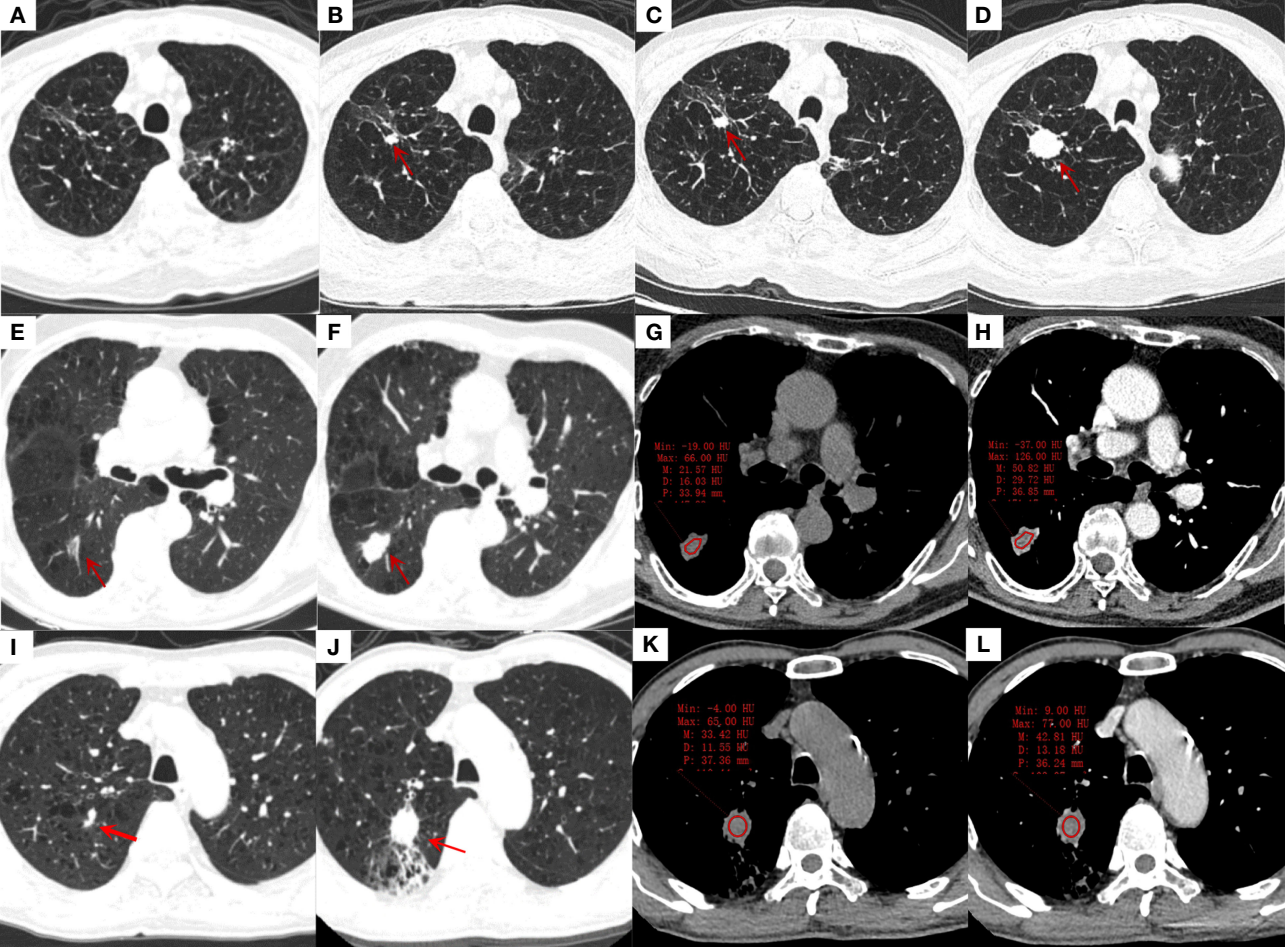
Transverse thin-section CT scans in the diagnosis of lung cancer in a lobe affected by tuberculosis. **(A-D)**, an 85-years-old male with stable tuberculosis; **(A)**: The first CT scan of the patient showed a few irregular linear and ground glass opacification in the right upper lung; **(B)**: CT scan was reexamined 4 years later, showing a new solid nodule (red arrow) with a diameter of 9 mm and lobulations in the original tuberculosis focus area; **(C, D)**: CT were reexamined 3 months and 8 months after the discovery of the nodule. The nodule was gradually enlarged, and small cell lung cancer was proved by bronchoscopic biopsy. **(E-H)**, an 82-years-old male with stable tuberculosis; **(E)**: a ground glass nodule (red arrow) with 16mm in length and vessel convergence was found in the dorsal segment of right lower lung on the first CT scan; **(F)**: the ground glass nodule was enlarged to solid nodule with 23mm in length, lobulations and spiculations after 4 years, adenocarcinoma was diagnosed by surgical biopsy. **(G, H)**: The non-enhanced and enhanced CT value of nodule was 22 HU and 51 HU, respectively; moderately heterogeneous enhancement can be seen after enhancement. **(I-L)**, a 68-years-old male with stable tuberculosis; **(I)**: a small solid nodule beside the vessel with 5 mm in length in the posterior segment of right upper lung was misdiagnosed as fibrous tubercle on the first CT scan; **(J)**: the nodule was obviously enlarged with 25 mm in length and distal obstructive pneumonia after 3 years, adenocarcinoma was diagnosed by surgical biopsy. **(K, L)**: The non-enhanced and enhanced CT value of nodule was 33 HU and 43 HU, respectively, ring-like enhancement can be seen after enhancement.

CT characteristics are shown in **Table 2**. The typical CT manifestations of three pulmonary tuberculosis patients who developed lung cancer were shown in **Figure 2**. Among the 41 patients with lung cancer, 17 (41%) were initially misdiagnosed as follows: failure to observe new nodules because of masking by coexisting stable tuberculosis lesions (n = 6), misdiagnosis as reactivation of tuberculosis (n = 5), failure to observe because of lesion subtleness (n = 4), and misdiagnosis as a common infection (n = 2). Compared to lesions in the control group, lesions in the lung cancer group were significantly more likely to demonstrate the following CT features which were defined as high-risk CT manifestations: large size, vessel convergence, lobulation, spiculation, spinous protuberance, bronchial obstruction or stenosis, vacuolation, ground-glass opacification, heterogeneous or homogeneous enhancement, and gradual increase in size (**Figure 2**). However, the control group was more likely to have a satellite lesion and had significantly higher minimum and mean CT values.

**Table 2:** CT features of patients with lung cancer occurrence in a lobe affected by tuberculosis and control group.

Project	Lung cancer group (n = 41)	Control group (n = 38)	*t/χ²* value	*P* value
Nodular diameter (mm)	26.5 ± 17.3	17.0 ± 12.1	2.828	0.006
Vessel convergence	24 (59%)	9 (24%)	9.724	0.002
Lobulations	33 (80%)	18 (47%)	9.335	0.002
Spiculations				
Long spiculations (length < 5 mm)	12 (29%)	10 (26%)	10.909	0.012
Short spiculations (length ≥ 5 mm)	14 (34%)	4 (11%)		
Long + Short spiculations	2 (5%)	0 (0%)		
Spiculation protuberance	16 (39%)	5 (13%)	6.676	0.010
Bronchial obstruction	19 (46%)	4 (11%)	12.103	< 0.001
Bronchial stenosis	18 (44%)	4 (11%)	10.795	0.001
Necrosis in nodule	4 (10%)	1 (3%)	1.667	0.197
Cavitation in nodule	2 (5%)	0 (0%)	1.878	0.171
Vacuolation in nodule	7 (17%)	0 (0%)	7.028	0.008
Calcification in nodule	18 (44%)	25 (66%)	3.761	0.053
Ground glass opacification	5 (12%)	0 (0%)	4.885	0.027
Degree of enhancement				
No enhancement	3 (3/32, 9%)	5 (5/12, 42%)	4.938	0.373
Mild enhancement	10 (10/32, 31%)	4 (4/12, 33%)		
Moderate enhancement	8 (8/32, 25%)	1 (1/12, 8%)		
High enhancement	11 (11/32, 34%)	2 (2/12, 17%)		
Enhancement patterns				
No enhancement	3 (3/32, 9%)	5 (5/12, 42%)	13.120	0.004
Heterogeneous enhancement	14 (14/32, 44%)	2 (2/12, 17%)		
Homogeneous enhancement	14 (14/32, 44%)	2 (2/12, 17%)		
Ring-like enhancement	1 (1/32, 3%)	3 (3/12, 25%)		
Minimum CT value of nodule (HU)	-40.0 ± 155.2	51.6 ± 202.1	2.247	0.028
Mean CT value of nodule (HU)	-14.9 ± 135.2	165.7 ± 312.8	3.286	0.002
Obstructive pneumonia	4 (4/41, 10%)	2 (2/38, 5%)	0.560	0.454
Obstructive atelectasis	2 (2/41, 5%)	1 (1/38, 3%)	0.269	0.604
Satellite lesions	11 (11/41, 27%)	25 (25/38, 66%)	11.916	<0.001
Mediastinal lymphadenectasis	19 (19/41, 46%)	15 (15/38, 39%)	0.722	0.395
Lymph node enhancement				
No enhancement	9 (9/32, 28%)	4 (4/12, 33%)	1.215	0.749
Homogeneous enhancement	21 (21/32, 66%)	8 (8/12, 67%)		
Ring-like enhancement	2 (2/32, 6%)	0 (0/12, 0%)		
Pleural thickening				
Nodular thickening	1 (1/41, 2%)	2 (2/38, 5%)	2.439	0.486
Diffuse thickening	1 (1/41, 2%)	0 (0/38, 0%)		
Uneven thickening	0 (0/41, 0%)	1 (1/38, 3%)		
No thickening	39 (39/41, 96%)	35 (35/38, 92%)		
Enlarged nodule at follow-up	37 (37/39, 95%)	0 (0/38, 0%)	70.485	< 0.001

### Diagnostic efficiency

As shown in **Table 3**, nodular enlargement showed the best diagnostic performance in the diagnosis of lung cancer in a lobe affected by tuberculosis (AUC = 0.974*; P <*0.001; accuracy = 98.2%; sensitivity = 94.7%; specificity = 100%). Mean nodule CT value (49 HU) also showed adequate diagnostic performance (AUC = 0.758*; P <*0.001; accuracy = 87.3%; sensitivity = 90.2%; specificity = 68.4%). The comparison of clinical and CT characteristics of patients with or without nodular enlargement and patients with high or low CT value (cut-off = 49 HU) are provided in the Online Appendix. Other clinical and CT features showed insufficient diagnostic performance (AUC <0.70).

**Table 3:** Diagnostic efficacy of clinical and CT features in patients with lung cancer occurrence in a lobe affected by tuberculosis and control group.

Index	AUC (95%CI)	*P* value	Optimal cut-off	Accuracy (%)	Sensitivity (%)	Specificity (%)
Elevated CEA	0.691 (0.530-0.824)	< 0.001		50.0	38.2	100
Nodular diameter	0.677 (0.563-0.778)	0.005	23.1 (mm)	41.8	58.5	81.6
Minimum CT value of nodule	0.614 (0.498-0.721)	0.084	28.8 (HU)	63.3	87.8	42.1
Mean CT value of nodule	0.758 (0.648-0.847)	< 0.001	49 (HU)	87.3	90.2	68.4
Vessel convergence	0.674 (0.560-0.775)	< 0.001		67.1	58.5	76.3
Lobulations	0.666 (0.551-0.768)	0.001		67.1	80.5	52.6
Spiculations	0.696 (0.582-0.794)	< 0.001		65.8	68.3	63.2
Spiculation protuberance	0.629 (0.513-0.735)	0.007		62.0	39.0	86.8
Bronchial obstruction	0.679 (0.565-0.780)	< 0.001		67.1	46.3	89.5
Bronchial stenosis	0.667 (0.552-0.769)	< 0.001		65.8	43.9	89.5
Vacuolation in nodule	0.573 (0.457-0.684)	0.009		57.0	17.1	100
Ground glass opacification	0.561 (0.445-0.673)	0.018		54.4	12.2	100
Enhancement patterns	0.557 (0.400-0.707)	0.640		77.3	90.6	41.7
Satellite lesions	0.695 (0.581-0.793)	< 0.001		30.4	26.8	34.2
Enlarged nodule at follow-up	0.974 (0.892-0.998)	< 0.001		98.2	94.7	100

## Discussion

At present many clinicians have insufficient understanding of lung cancer affected by stable pulmonary tuberculosis, so the CT re-examination of patients with stable pulmonary tuberculosis is mainly carried out according to the consensus and guidelines of tuberculosis for evaluation of treatment response (10, 11) instead of low-dose CT (LDCT) guidelines for lung cancer screening (9, 12). At the same time, many tuberculosis patients did not have regular CT re-examination according to the clinician’s recommendation because they had no symptoms of tuberculosis and wanted to save cost. These reasons lead to the unequal interval of CT re-examination and frequent delays in the diagnosis of lung cancer. Among the 41 lung cancer patients with stable pulmonary tuberculosis in our study, 41% (17/41) were initially misdiagnosed. The main reason for these misdiagnoses may have been failure to observe new nodules because of masking by stable tuberculosis lesions. Since tuberculosis can also manifest as pulmonary nodules, early diagnosis of lung cancer can be difficult, which can delay treatment and affect patient prognosis (13).

CEA is a non-specific tumor marker which cannot be used as a diagnostic marker for lung cancer (14). It is mainly used for the observation of the effect of operation and chemotherapy, and can also be used for the prognostic evaluation of lung cancer patients (14). However, the obvious increase or continuous increase of CEA level should be paid more attention to in clinic (14). Adenocarcinoma was the most common pathological type in our lung cancer group (68%), which was similar to earlier studies (15, 16). Meanwhile, adenocarcinoma is the most common type of lung cancer with elevated CEA levels, which may explain the higher CEA levels in our group of lung cancer patients than in the control group. A Positive result of TSPOT shows the presence of Mycobacterium tuberculosis infection including active and latent tuberculosis infection (LTBI), which are more specific than the tuberculin skin test (TST) for diagnosis of LTBI (17). We found that 71% of patients in the lung cancer group were TSPOT positive, but the difference was not statistically significant compared with the control group. Similar results were obtained from the TST, indicating that there were other causes besides tuberculosis involved in lung cancer occurrence of tuberculosis patients. A previous population cohort study has found that 1/4 of newly diagnosed lung cancer patients have LTBI, and the tumors are mostly distributed in the location of tuberculosis infection (18). The coexistence of tuberculosis with bronchogenic carcinomas was detected in 30.2% of necropsy cases of bronchogenic carcinomas (19), while the incidence of tuberculosis amongst the general autopsies performed in the same department does not exceed 7-8%. The main pathological type of lung cancer associated with scarring is adenocarcinoma (20), and similar pathological findings were obtained in our study. Previous studies have reported that the risk of lung cancer is higher in tuberculosis patients than in those without tuberculosis (18, 21, 22). Chronic inflammatory stimulation, immune abnormalities, and gene mutations caused by tuberculosis infection and anti-tuberculosis drugs may be associated to the occurrence of lung cancer. Firstly, inflammation and pulmonary fibrosis caused by tuberculosis may lead to genetic damage that increases the risk of lung cancer (23–25). Secondly, tuberculosis may weaken the immune system and promote tumorigenesis (15). Thirdly, scar tissue may lead to atypical metaplasia and early peripheral pulmonary carcinoma can originate from terminal bronchioles around the scar (20). Fourthly, isoniazid was included in the list of Group 3 carcinogens by the international agency for research on cancer (IARC) of the World Health Organization, but more in-depth research is needed on whether anti-tuberculosis drugs can cause lung cancer. At the same time, detailed studies of molecular mechanism of tuberculosis infection in promoting lung cancer, as well as, for collection of more epidemiologic data are necessary.

It is not easy to distinguish lung cancer complicating tuberculosis from stable tuberculosis alone on CT. In this study, frequency of vessel convergence, lobulation, spiculation, and spinous protuberance was significantly higher in the lung cancer group than the control group and intranodular vacuoles were found only in the lung cancer group. These findings are considered classical CT features of peripheral lung cancer (26–28) and consistent with the recommendations of the Fleischner society (7, 12). The incidence of bronchial stenosis or obstruction was also significantly higher in the lung cancer group than the control group, probably because tumors of bronchial origin infiltrated submucosally to cause narrowing of the bronchia (29). In this study, ground-glass opacification of 5 (12%) patients who had an adenocarcinoma were mistaken for the exudation and fibrosis caused by tuberculosis (30). We suggest that patients with stable pulmonary tuberculosis exhibiting ground-glass opacification should be closely followed with early lung cancer screening. This retrospective study found that 95% of lung cancer patients had nodular enlargement at follow-up. Unfortunately, the interval between CT re-examination varied due to the lack of awareness of lung cancer affected by tuberculosis by both clinicians and patients, and many patients didn’t come to the hospital for CT re-examination until they had lung cancer symptoms, resulting in delay in diagnosis and treatment. The increased nodular diameter during follow-up had the best diagnostic performance, which further indicated the importance of regular CT re-examination. Therefore, we suggest that middle-aged and elderly patients with stable pulmonary tuberculosis should undergo low-dose CT annually, and patients with high-risk features on initial CT should be re-examined within 3–6 months to avoid delayed diagnosis.

Non-enhanced CT attenuation values were noticeably higher in stable tuberculosis nodules, which are mainly composed of fibrosis and calcification, than values in lung cancer nodules in our study (*P* = 0.002). Diagnostic sensitivity, specificity, and accuracy were 68.4%, 90.2%, and 87.3%, respectively, using 49 HU as the cut-off. Therefore, lung cancer should be considered in lesions with a CT attenuation value lower than 49 HU. Our study also found that lung cancer nodules mainly enhanced heterogeneously or homogeneously, while tuberculosis nodules did not enhance or exhibited ring-like enhancement, which is consistent with previous studies (31). Mean non-enhanced CT attenuation values and enhancement patterns of nodules should be considered together when attempting to discover lung cancer in patients with stable pulmonary tuberculosis.

Many clinical guidelines recommend FDG PET/CT for the evaluation of noncalcified pulmonary nodules ≥8 mm detected during low dose computed tomography (LDCT) lung cancer screening, and the use of FDG PET for the evaluation of ground-glass opacities was discouraged (9, 12, 32). In our case series, only 9(22%) patients underwent PET/CT, while none of the control group underwent PET/CT. It is noteworthy that all the patients went for PET/CT because the suspected malignant lesions found by CT. In pulmonary tuberculosis, inflammatory cells typically demonstrate increased ¹⁸F-FDG uptake, and areas of active tuberculosis can be differentiated from old or inactive disease (33). However, standardized uptake value measurements are high in both tuberculosis and malignant lesions, with significant overlap that limits their usefulness (33, 34). As the differentiation of malignant lesions versus tuberculosis involvement is problematic and also the high radiation dose and high cost of PET/CT, it may be not suitable as a basic screening method for lung cancer in patients with tuberculosis.

This study has several limitations. First, it was retrospective in design, conducted in a single center, and had a sample size. Second, some patients did not undergo continuous follow-up and the interval between CT re-examination varied; therefore, our findings should be interpreted as descriptive and preliminary. Next a prospective multicenter study to observe the accurate temporal characteristics of lung cancer in a lobe affected by stable pulmonary tuberculosis is needed. Third, some cases didn’t undergo contrast enhanced CT-scans and dynamic enhanced scanning was not performed which precluded time-density curve analysis of nodules in the control group, which may have affected the results of the enhancement characteristics analysis. Fourth, the clinical utility of FDG PET/CT during the evaluation of lung nodules detected on CT need further evaluation. We hope that the preliminary results of this study will stimulate further researches in this area.

In conclusion, patients with stable pulmonary tuberculosis represent a group at risk for developing lung cancer and have to be controlled continuously. Regular CT re-examination is necessary and further prospective multicenter studies are needed to help guide the lung cancer screening of patients with stable pulmonary tuberculosis.

## Data availability statement

The raw data supporting the conclusions of this article will be made available by the authors, without undue reservation.

## Ethics statement

The studies involving human participants were reviewed and approved by Ethic Committee of the Xiangya Hospital of Central South University. The patients/participants provided their written informed consent to participate in this study. Written informed consent was obtained from the individual(s) for the publication of any potentially identifiable images or data included in this article.

## Author contributions

HZ conceived and designed the research. KL, LL acquired the data. HZ, KL analyzed and interpreted the data. HZ, KL and JC performed statistical analysis. KL and YL drafted the manuscript. HZ made critical revision of the manuscript for important intellectual content. All authors contributed to the article and approved the submitted version.

## Funding

This work was supported by Natural Science Foundation of Hunan Province, China (2021JJ31131).

## Conflict of interest

The authors declare that the research was conducted in the absence of any commercial or financial relationships that could be construed as a potential conflict of interest.

## Publisher’s note

All claims expressed in this article are solely those of the authors and do not necessarily represent those of their affiliated organizations, or those of the publisher, the editors and the reviewers. Any product that may be evaluated in this article, or claim that may be made by its manufacturer, is not guaranteed or endorsed by the publisher.
