# Supplementary material for: The value of chest computed tomography in evaluating lung cancer in a lobe affected by stable pulmonary tuberculosis in middle-aged and elderly patients: A preliminary study
Source: Front Oncol. 2022 Oct 6;12:868107. doi: 10.3389/fonc.2022.868107 (PMC9582123; doi:10.3389/fonc.2022.868107)
Supplement: Supplementary file 1 [file Table_1.docx]

**Supplementary material**

**Table 1:** Clinical and CT characteristics of patients with or without nodular enlargement

| Project | enlargement group  (n=39) | no enlargement group  (n=40) | *t* / *χ²* value | *P* value |
| --- | --- | --- | --- | --- |
| Age (years) | 66.9±8.5 | 65.3±11.9 | 0.451 | 0.504 |
| Sex  Male  Female | 33 (85%)  6 (15%) | 28 (70%)  12 (30%) | 2.367 | 0.124 |
| Years of tuberculosis | 16.9±15.3 | 11.9±11.9 | 1.244 | 0.272 |
| Smoking | 25 (64%) | 20 (50%) | 1.582 | 0.209 |
| Diabetes | 3 (8%) | 5 (13%) | 0.495 | 0.482 |
| CEA, elevated | 12 (12/33, 36%) | 1 (1/9, 11%) | 2.060 | 0.151 |
| NSE, elevated | 1 (1/32, 3%) | 1 (1/23, 4%) | 0.056 | 0.813 |
| CA-125, elevated | 3 (3/23, 13%) | 1 (1/15, 7%) | 0.382 | 0.537 |
| SCC, elevated | 1 (1/18, 6%) | 0 (0/10, 0%) | 0.556 | 0.456 |
| NSCLC-21-1, elevated | 3 (3/17, 18%) | 0 (0/10, 0%) | 1.912 | 0.167 |
| CK19, elevated | 4 (4/32, 13%) | 1 (1/16, 6%) | 0.437 | 0.509 |
| Tuberculosis antibody, positive | 3 (3/26, 11%) | 4 (4/19, 21%) | 3.927 | 0.140 |
| TSPOT, positive | 19 (19/26, 73%) | 11 (11/14, 79%) | 0.143 | 0.706 |
| Nodular diameter (mm) | 25.9±16.2 | 18.1±14.2 | 5.158 | 0.026 |
| Vessel convergence | 24(62%) | 9(23%) | 12.216 | ＜0.001 |
| Lobulations | 32(82%) | 19(48%) | 10.172 | 0.001 |
| Spiculations |  |  |  |  |
| Long spiculations (length < 5 mm) | 12(31%) | 10(25%) | 10.294 | 0.016 |
| Short spiculations (length ≥ 5 mm) | 13(33%) | 5(13%) |  |  |
| Long + Short spiculations | 2(5%) | 0(0%) |  |  |
| Spiculation protuberance | 16(39%) | 5(13%) | 8.129 | 0.004 |
| Bronchial obstruction | 18(46%) | 5(13%) | 10.7 | 0.001 |
| Bronchial stenosis | 17(44%) | 5(13%) | 9.379 | 0.002 |
| Necrosis in nodule | 3(8%) | 2(5%) | 0.238 | 0.625 |
| Cavitation in nodule | 2(5%) | 0(0%) | 2.078 | 0.149 |
| Vacuolation in nodule | 6(15%) | 1(3%) | 4.008 | 0.045 |
| Calcification in nodule | 17(44%) | 26(65%) | 3.603 | 0.058 |
| Ground glass opacification | 6(15%) | 0(0%) | 6.575 | 0.010 |
| Degree of enhancement |  |  |  |  |
| No enhancement | 5(5/30, 17%) | 5(5/14, 36%) | 2.376 | 0.498 |
| Mild enhancement | 8(8/30, 27%) | 4(4/14, 29%) |  |  |
| Moderate enhancement | 7(7/30, 23%) | 2(2/14, 14%) |  |  |
| High enhancement | 10(10/30, 33%) | 3(3/14, 21%) |  |  |
| Enhancement patterns |  |  |  |  |
| No enhancement | 3(3/30, 10%) | 5(5/14, 36%) | 16.055 | 0.001 |
| Heterogeneous enhancement | 14(14/30, 47%) | 2(2/14, 14%) |  |  |
| Homogeneous enhancement | 13(13/30, 43%) | 3(3/14, 21%) |  |  |
| Ring-like enhancement | 0(0/30, 0%) | 4(4/14, 29%) |  |  |
| Minimum CT value of nodule (HU) | -43.8±158.2 | 50.8±196.9 | 5.514 | 0.021 |
| Mean CT value of nodule (HU) | -17.9±138.0 | 159.6±305.9 | 10.953 | 0.001 |
| Obstructive pneumonia | 3(3/39, 10%) | 3(3/40, 5%) | 0.001 | 0.974 |
| Obstructive atelectasis | 2(2/39, 5%) | 1(1/40, 3%) | 0.317 | 0.574 |
| Satellite lesions | 11(11/39, 27%) | 25(25/40, 66%) | 9.245 | 0.002 |
| Mediastinal lymphadenectasis | 18(18/39, 46%) | 15(15/40, 39%) | 0.600 | 0.439 |
| Lymph node enhancement |  |  |  |  |
| No enhancement | 9(9/31, 29%) | 5(4/14, 33%) | 1.280 | 0.734 |
| Homogeneous enhancement | 21(21/31, 66%) | 8(8/14, 67%) |  |  |
| Ring-like enhancement | 1(2/31, 6%) | 1(1/14, 7%) |  |  |
| Pleural thickening |  |  |  |  |
| Nodular thickening | 1(1/39, 2%) | 2(2/40, 5%) | 2.321 | 0.509 |
| Diffuse thickening | 1(1/39, 2%) | 0(0/40, 0%) |  |  |
| Uneven thickening | 1(1/39, 2%) | 1(1/40, 3%) |  |  |
| No thickening | 37(37/39, 96%) | 37(37/40, 93%) |  |  |
| Lung cancer | 39(39/39, 100%) | 2(2/40, 5%) | 70.485 | ＜0.001 |

**Table 2:** Clinical and CT characteristics of patients with high or low CT value (cut-off = 49 HU)

| Project | low CT value group  (n=49) | high CT value group  (n=30) | *t* / *χ²* value | *P* value |
| --- | --- | --- | --- | --- |
| Age (years) | 67.3±8.9 | 64.0±12.2 | 1.901 | 0.172 |
| Sex  Male  Female | 39 (80%)  10 (20%) | 22 (73%)  8 (27%) | 0.409 | 0.522 |
| Years of tuberculosis | 14.5±15.0 | 13.4±10.9 | 0.059 | 0.810 |
| Smoking | 29(59%) | 16(53%) | 0.256 | 0.613 |
| Diabetes | 5(10%) | 3 (10%) | 0.001 | 0.977 |
| CEA, elevated | 12 (12/33, 36%) | 1 (1/9, 11%) | 2.060 | 0.151 |
| NSE, elevated | 1 (1/36, 3%) | 1 (1/19, 4%) | 0.215 | 0.643 |
| CA-125, elevated | 3 (3/26, 12%) | 1 (1/12, 8%) | 2.009 | 0.156 |
| SCC, elevated | 1 (1/19, 5%) | 0 (0/9, 0%) | 0.474 | 0.491 |
| NSCLC-21-1, elevated | 3 (3/18, 17%) | 0 (0/9, 0%) | 1.625 | 0.202 |
| CK19, elevated | 4 (4/33, 12%) | 1 (1/15, 7%) | 0.322 | 0.570 |
| Tuberculosis antibody, positive | 5 (5/28, 18%) | 2 (2/17, 12%) | 3.3.589 | 0.166 |
| TSPOT, positive | 21 (21/27, 78%) | 9 (9/13, 69%) | 0.333 | 0.564 |
| Nodular diameter (mm) | 24.8±16.8 | 17.2±12.5 | 4.676 | 0.034 |
| Vessel convergence | 25(51%) | 8(23%) | 4.480 | 0.034 |
| Lobulations | 36(73%) | 15(48%) | 4.423 | 0.036 |
| Spiculations |  |  |  |  |
| Long spiculations (length < 5 mm) | 14(29%) | 8(27%) | 3.041 | 0.385 |
| Short spiculations (length ≥ 5 mm) | 13(27%) | 5(17%) |  |  |
| Long + Short spiculations | 2(4%) | 0(0%) |  |  |
| Spiculation protuberance | 16(33%) | 5(17%) | 2.406 | 0.121 |
| Bronchial obstruction | 19(39%) | 4(13%) | 5.762 | 0.016 |
| Bronchial stenosis | 19(39%) | 3(10%) | 7.571 | 0.006 |
| Necrosis in nodule | 5(10%) | 0(0%) | 3.227 | 0.072 |
| Cavitation in nodule | 1(2%) | 1(3%) | 0.124 | 0.724 |
| Vacuolation in nodule | 5(10%) | 2(7%) | 0.285 | 0.594 |
| Calcification in nodule | 20(41%) | 23(77%) | 9.520 | 0.002 |
| Ground glass opacification | 6(12%) | 0(0%) | 3.925 | 0.048 |
| Degree of enhancement |  |  |  |  |
| No enhancement | 6(6/34, 17%) | 4(4/10, 40%) | 3.409 | 0.333 |
| Mild enhancement | 9(9/34, 26%) | 3(3/10, 30%) |  |  |
| Moderate enhancement | 7(7/34, 21%) | 2(2/10, 20%) |  |  |
| High enhancement | 12(12/34, 35%) | 1(1/10, 10%) |  |  |
| Enhancement patterns |  |  |  |  |
| No enhancement | 5(5/34, 15%) | 3(3/10, 30%) | 5.209 | 0.157 |
| Heterogeneous enhancement | 15(15/34, 44%) | 1(1/10, 10%) |  |  |
| Homogeneous enhancement | 12(12/34, 35%) | 4(4/10, 40%) |  |  |
| Ring-like enhancement | 2(2/34, 6%) | 2(2/10, 20%) |  |  |
| Obstructive pneumonia | 5(5/49, 10%) | 1(1/30, 3%) | 1.236 | 0.266 |
| Obstructive atelectasis | 1(1/49, 2%) | 2(2/30, 7%) | 1.076 | 0.300 |
| Satellite lesions | 22(22/49, 45%) | 14(14/30, 47%) | 0.023 | 0.879 |
| Mediastinal lymphadenectasis | 23(23/49, 47%) | 10(10/30, 33%) | 1.398 | 0.237 |
| Lymph node enhancement |  |  |  |  |
| No enhancement | 8(8/35, 23%) | 5(5/10, 50%) | 3.257 | 0.354 |
| Homogeneous enhancement | 24(24/35, 69%) | 5(5/10, 50%) |  |  |
| Ring-like enhancement | 2(2/35, 6%) | 0(0/10, 0%) |  |  |
| Pleural thickening |  |  |  |  |
| Nodular thickening | 2(2/49, 4%) | 1(1/30, 3%) | 2.274 | 0.518 |
| Diffuse thickening | 1(1/49, 2%) | 0(0/30, 0%) |  |  |
| Uneven thickening | 0(0/49, 0%) | 1(1/30, 3%) |  |  |
| No thickening | 46(46/49, 94%) | 28(28/30, 93%) |  |  |
| Lung cancer | 37(37/49, 76%) | 4(4/30, 13%) | 28.451 | ＜0.001 |
